# Galectin-9 Enhances Cytokine Secretion, but Suppresses Survival and Degranulation, in Human Mast Cell Line

**DOI:** 10.1371/journal.pone.0086106

**Published:** 2014-01-20

**Authors:** Reiji Kojima, Tatsukuni Ohno, Motoyasu Iikura, Toshiro Niki, Mitsuomi Hirashima, Keichi Iwaya, Hitoshi Tsuda, Shigeaki Nonoyama, Akio Matsuda, Hirohisa Saito, Kenji Matsumoto, Susumu Nakae

**Affiliations:** 1 Department of Basic Pathology, National Defense Medical College, Saitama, Japan; 2 Department of Pediatrics, National Defense Medical College, Saitama, Japan; 3 Department of Allergy and Immunology, National Research Institute for Child Health and Development, Tokyo, Japan; 4 Department of Respiratory Medicine, National Center for Global Health and Medicine, Tokyo, Japan; 5 Departments of Immunology and Immunopathology, Faculty of Medicine, Kagawa University, Takamatsu, Japan; 6 Research Center, GalPharma Company, Takamatsu, Japan; 7 Laboratory of Systems Biology, Center for Experimental Medicine and Systems Biology, The Institute of Medical Science, The University of Tokyo, Tokyo, Japan; 8 Precursory Research for Embryonic Science and Technology (PRESTO), Japan Science and Technology Agency, Saitama, Japan; French National Centre for Scientific Research, France

## Abstract

Galectin-9 (Gal-9), a lectin having a β-galactoside-binding domain, can induce apoptosis of Th1 cells by binding to TIM-3. In addition, Gal-9 inhibits IgE/Ag-mediated degranulation of mast cell/basophilic cell lines by binding to IgE, thus blocking IgE/Ag complex formation. However, the role of Gal-9 in mast cell function in the absence of IgE is not fully understood. Here, we found that recombinant Gal-9 directly induced phosphorylation of Erk1/2 but not p38 MAPK in a human mast cell line, HMC-1, which does not express FcεRI. Gal-9 induced apoptosis and inhibited PMA/ionomycin-mediated degranulation of HMC-1 cells. On the other hand, Gal-9 induced cytokine and/or chemokine production by HMC-1 cells, dependent on activation of ERK1/2 but not p38 MAPK. In addition, the lectin activity of Gal-9 was required for Gal-9-mediated cytokine secretion by HMC-1 cells. These observations suggest that Gal-9 has dual properties as both a regulator and an activator of mast cells.

## Introduction

Galectin-9 (Gal-9) was first identified as a chemoattractant and activating factor for eosinophils. [1]–[3] It is abundantly expressed in various tissues, especially the epithelium of the gastrointestinal tract, and in a variety of cells such as macrophages, eosinophils, mast cells, fibroblasts and synovial cells. [4]–[7].

Gal-9 influences various biological functions such as cell aggregation, adhesion, apoptosis, survival, activation and differentiation by binding to T-cell immunoglobulin and mucin domain-containing protein 3 (TIM-3). [5], [8], [9] Like Gal-9, Tim-3 is also expressed on various types of cells, including Th1 cells, [9] Tc1 cells, [10] Th17 cells, [11] NK cells, [12] NKT cells, [13], [14] dendritic cells (DC) [15] and mast cells (MCs). [16], [17] It is known that Gal-9 has anti-tumor activity by promoting activation of NK cells [18] and cytotoxic T lymphocytes by enhancing DC maturation. [19] Moreover, Gal-9 induces aggregation of melanoma and breast cancer cell lines and suppresses metastasis. [20]–[22] It was suggested that Gal-9 is a negative regulator of development of autoimmune diseases such as experimental autoimmune encephalomyelitis (EAE) and collagen-induced arthritis (CIA) in mice. Indeed, like anti-TIM-3 mAb, [10] Gal-9 can suppress development of EAE by inducing Th1 cell apoptosis via TIM-3. [9] Gal-9 can also attenuate development of CIA by inhibiting differentiation of Th17 cells while enhancing differentiation of regulatory T cells. [23] Moreover, expression of each of Gal-9 and TIM-3 was shown to be increased in the lungs of rodents during allergic airway inflammation, [24]–[26] suggesting roles for Gal-9 and TIM-3 in induction of that disease. Indeed, Gal-9 administration to mice suppressed ovalbumin- and house dust mite antigen-induced airway inflammation and hypersensitivity. [27] In the setting, Gal-9 bound to CD44, interfering with binding of hyaluronan, a known ligand for CD44, and resulting in inhibition of Th2 cell recruitment through CD44-hyaluronan interaction. [27] On the other hand, the role of TIM-3 in development of ovalbumin-induced airway inflammation and hypersensitivity is controversial. That is, the response was attenuated in mice treated with anti-TIM-3 mAb, [24] but normal in TIM-3-deficient mice. [28] Although the reason for that apparent discrepancy is unclear, the report using anti-TIM-3 mAbs did not fully characterize them, i.e., whether they were agonistic, blocking or depletion Abs. These observations suggest that the biological function of Gal-9 may be mediated independently of TIM-3 in certain settings. In support of this, binding of Gal-9 to IgE blocks IgE/Ag complex formation and thus inhibits IgE/Ag-FcεRI crosslinking-induced degranulation of mast cell/basophilic cell lines. [29] In contrast, we showed that anti-TIM-3 agonistic antibody promoted cytokine secretion, but did not influence degranulation, by mouse bone marrow cell-derived cultured mast cells (BMCMCs) after IgE/Ag-FcεRI crosslinking. [16] On the other hand, the role of Gal-9 in mast cell function in the absence of IgE remains unclear. Therefore, in the present study we examined the role of Gal-9 in the functions of a human mast cell line, HMC-1, which does not express FcεRI, in the absence of IgE/Ag stimulation. We found that human Gal-9 enhanced cytokine secretion, but suppressed survival and degranulation, of HMC-1. These observations suggest that Gal-9 has dual properties as a regulator and activator of mast cells.

## Materials and Methods

### Cell Culture

HMC-1 cells (a human mast cell line) [30] were cultured in α-minimum essential medium (Gibco BRL, Grand Island, NY, USA) supplemented with 10% FBS, 100 U/ml penicillin and 100 µg/ml streptomycin under a humidified atmosphere of 5% CO_2_ at 37°C. Half of the medium was replaced twice per week. Normal human bronchial epithelial cells (NHBEs), normal human coronary artery endothelial cells (HCAECs) and normal human lung fibroblasts (NHLF) were obtained from Lonza (Wakersville, MD, USA) and were cultured as described elsewhere. [31].

### Quantitative PCR

Total RNA samples were isolated from HMC-1 cells, NHBEs and HCAECs using RNeasy (Qiagen, Valencia, CA, USA) and digested with RNase-free DNase I (Qiagen) in accordance with the manufacturer’s instructions. Human universal reference (HUR) RNA (BD Biosciences, Palo Alto, CA, USA) was used as a positive control. Then first-strand cDNA was synthesized from the isolated RNA using an iScript cDNA Synthesis Kit (Bio-Rad, Hercules, CA, USA). Primers for TIMs and GAPDH were designed as follows: TIM-1 (sense, 5′-TGT TCC TCC AAT GCC TTT GC-3′; antisense, 5′-TTG CTC CCT GCA GTG TCG TA-3′), TIM-3 (sense, 5′-CAA TGC CAT AGA TCC AAC CAC C-3′; antisense, 5′-GCA GTG GAC AGA ACC TCC AAA A-3′), TIM-4 (sense, 5′-TCC TGC TGA CAT CCA AAG CA-3′; antisense, 5′-TGG GAG ATG GGC ATT TCA TT-3′) and GAPDH (sense, 5′-GAA GGT GAA GGT CGG AGT C-3′; antisense, 5′-GAA GAT GGT GAT GGG ATT TC-3′). To determine the exact copy numbers of the target genes, quantified concentrations of the purified PCR products of TIM-1, TIM-3, TIM-4 and GAPDH were serially diluted and used as standards in each experiment. Aliquots of cDNA equivalent to 5 ng of the total RNA samples were used for each quantitative PCR. The mRNA expression levels were normalized to the GAPDH level in each sample.

### Flow Cytometry

Cells (HMC-1 cells and PBMCs) were incubated with human AB serum (Lonza) at 4°C for 5 min, and then stained with PE-conjugated anti-human TIM-1 mAb (Clone Name 1D12, BioLegend, San Diego, CA, USA), PE-conjugated anti-human TIM-3 mAb (Clone Name F38-2E2, BioLegend), PE-conjugated anti-human TIM-4 mAb (Clone Name 9F4, BioLegend) and PE-conjugated mouse IgG1 (Clone Name MOPC-21, BioLegend) at 4°C for 30 min. The expression of TIMs on the cells was determined with a FACS Canto II using Diva Software (BD Biosciences, San Jose, CA, USA).

### Western Blotting

HMC-1 cells (5×10^5^ cells/well in a 24-well plate) were treated with 1 µM recombinant human galectin-9 (rhGal-9) (GalPhama Co., Ltd., Kagawa, Japan) at 37°C for the indicated time periods. Then the cells were lysed and sonicated in 200 µl of NuPAGE sample buffer (Invitrogen, Carlsbad, CA, USA) containing 5% 2-mercaptoethanol. Proteins in the whole-cell lysates were separated by SDS-PAGE (5–15% Ready Gels J; Bio-Rad) gel electrophoresis and transferred to nitrocellulose membranes (iBlot Gel Transfer Stacks, mini; Invitrogen). Immunoblotting was performed using rabbit anti-phospho-p44/42 MAPK (Erk1/2) mAb (clone D13.14.4E; Cell Signaling Technology, Danvers, MA) and rabbit anti-p44/42 MAPK (Erk1/2) mAb (clone 137F5; Cell Signaling Technology) as the 1^st^ Abs and horseradish peroxidase-conjugated anti-rabbit IgG (Cell Signaling Technology) as the 2^nd^ Ab. The protein bands were visualized by enhanced Pierce Western Blotting Substrate (Thermo Scientific, Rockford, IL, USA).

### Cell Survival

HMC-1 cells were pretreated with or without 5 µg/ml mitomycin-C (Sigma-Aldrich Chemical Co., St. Louis, MO, USA) for 2 hours. After washing, the cells (4×10^5^ cells/ml) were cultured in the presence and absence of 0.25, 0.5 and 1 µM rhGal-9 at 37°C for 0, 24 or 48 h. Live cells were counted under a microscope after trypan blue staining. The cells were incubated with FITC-conjugated annexin V and propidium iodide (MEBCYTO-Apoptosis Kit; MBL Co., Ltd., Nagoya, Japan), and the percentage of propidium iodide-negative and annexin V-positive apoptotic cells was determined using a FACSCanto II with Diva Software.

### Caspase Activity

HMC-1 cells (1×10^5^ cells/ml) were cultured in the presence and absence of 0.5 µM rhGal-9 or 0.1 µM staurosporine (Cayman Chemical Company, Ann Arbor, MI, USA) at 37°C for 16 hours. Then the caspase-3/7 activities in the cells were determined by Caspase-Glo 3/7 assay (Promega, Madison, WI, USA) in accordance with the manufacturer’s instructions. The luminescence (Relative Light Unit [RLU]) of each sample was measured with a fluorescence plate reader (ARVO X5, PerkinElmer, Waltham, MA, USA) at 490/535 nm.

### β-hexosaminidase Release Assay

HMC-1 cells (1×10^5^ cells/well in a 96-well plate; not treated with mitomycin C) were pre-treated with rhGal-9 (0, 0.25, 0.5 and 1 µM) at 37°C for 30 min, and then stimulated with 0.1 µg/ml PMA (Sigma Chemical Co.) and 1 µg/ml ionomycin (Sigma Chemical Co.) at 37°C for 30 min. The culture supernatants were collected, and the activity of β-hexosaminidase in each was determined as described previously, with minor modification. [32] In brief, 50-µl samples were incubated with 100 µl of 1.3 mg/ml p-nitrophenyl-N-acetyl-β-D-glucosaminide (Sigma Chemical Co.) in 0.1 M sodium citrate (pH 4.5) in a 96-well microtiter plate at 37°C for 1 h. The enzymatic reaction was stopped by addition of 50 µl of 0.4 M glycine (pH 10.7) to each well. Enzymatic activities (OD405) were measured using a plate reader. Data show the percent release of β-hexosaminidase under various conditions of stimulation relative to the total amount of β-hexosaminidase in the cells, as measured in the supernatants of frozen and thawed cells.

### ELISA

HMC-1 cells (1×10^5^ cells/well in a 96-well plate) were treated with various concentrations of rhGal-9 in the presence and absence of 20 mM lactose (Nacalai Tesque, Kyoto, Japan) or sucrose (Wako, Osaka, Japan) at 37°C for 18 h. In some cases, HMC-1 cells were treated with ERK inhibitor (PD98059; Calbiochem, La Jolla, CA, USA), ERK inhibitor control (SB202474; Calbiochem) or solvent (0.1% (v/v) DMSO) alone at 37°C for 30 min, and with rhTIM-3-Fc (R&D Systems, Minneapolis, MN, USA) or human IgG (Sigma Chemical Co.) at 37°C for 1 hour before exposure to rhGal-9. The levels of IL-6, IL-8 and MCP-1 in the culture supernatants were determined with ELISA kits (R&D Systems) in accordance with the manufacturer’s instructions.

### Statistics

All data are expressed as means ± SD. The unpaired Student’s t-test, two-tailed, or ANOVA, as appropriate, was used for statistical evaluation of the results. *P*<0.05 was considered statistically significant.

## Results

### Expression of TIM Family Members’ mRNA in Human Mast Cell Line

**Figure 1:**
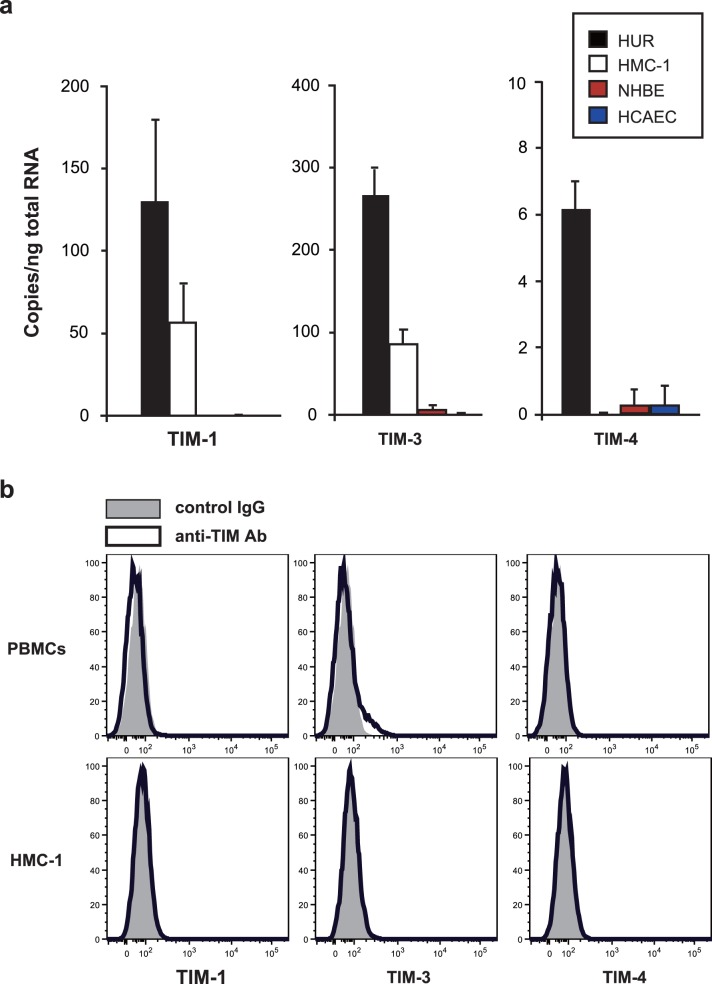
Expression of TIM family members in cultured human mast cell line and primary cells. (a) The mRNA expression for TIM family members (TIM-1, TIM-3 and TIM-4) in a human mast cell line, HMC-1, normal human bronchial epithelial cells (NHBEs) and normal human coronary artery endothelial cells (HCAECs) was determined by quantitative PCR. Human universal reference (HUR) RNA was used as a control. (b) The cell surface expression of TIM family members (TIM-1, TIM-3 and TIM-4) on HMC-1 cells and PBMCs was determined by flow cytometry. Shaded areas = isotype-matched control IgG staining, and bold lines = anti-TIM mAb staining. Data show a representative result of two independent experiments.

As in our earlier study using mouse mast cells (16), we first examined the expression of mRNA for TIM family members (TIM-1, TIM-3 and TIM-4) in HMC-1 cells and other human primary cells (NHBE and HCAEC, as negative controls) by quantitative PCR. We found constitutive expression of mRNA for both TIM-1 and TIM-3 in HMC-1 cells, but not in NHBE or HCAEC (Figure 1a). On the other hand, expression of TIM-4 mRNA was barely detectable in these cells. Next, we determined the surface protein expression of the TIM family members (TIM-1, TIM-3 and TIM-4) in HMC-1 cells and PBMCs by flow cytometry. In contrast to mRNA expression, TIM-1 and TIM-3 as well as TIM-4 were barely detectable on either HMC-1 cells or PBMCs (Figure 1b).

### Gal-9 Induces Phosphorylation of Erk1/2 in HMC-1 Cells

**Figure 2:**
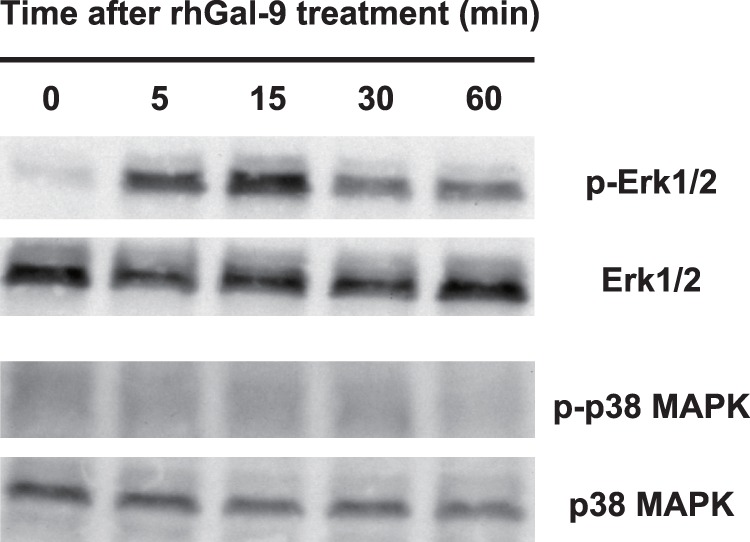
Galectin-9 induces phosphorylation of Erk1/2, but not p38 MAPK, in HMC-1 cells. HMC-1 cells were cultured in the presence of 1 µM recombinant human galectin-9 (rhGal-9) for the indicated times. Then the levels of phosphorylation of Erk1/2 and p38 MAPK in the cells were determined by western blot analysis. Data show a representative result of three independent experiments.

We previously demonstrated that IL-33 can induce cytokine secretion by human mast cells, although surface expression of ST2, a component of IL-33R, is barely detectable on these cells by flow cytometry [33]. Likewise, although surface expression of TIM-3 is barely detectable on HMC-1 cells, Gal-9, which is a ligand for TIM-3, may play some role in activation and/or regulation of HMC-1 cells. Thus, we examined the effect of rhGal-9 on the phosphorylation of signaling molecules in those cells by immunoblot analysis. We found that rhGal-9 induced phosphorylation of Erk1/2, but not p38 MAPK, in HMC-1 cells (Figure 2), suggesting that rhGal-9 may influence the function of HMC-1 cells.

### Gal-9 Induces Apoptosis of HMC-1 Cells

**Figure 3:**
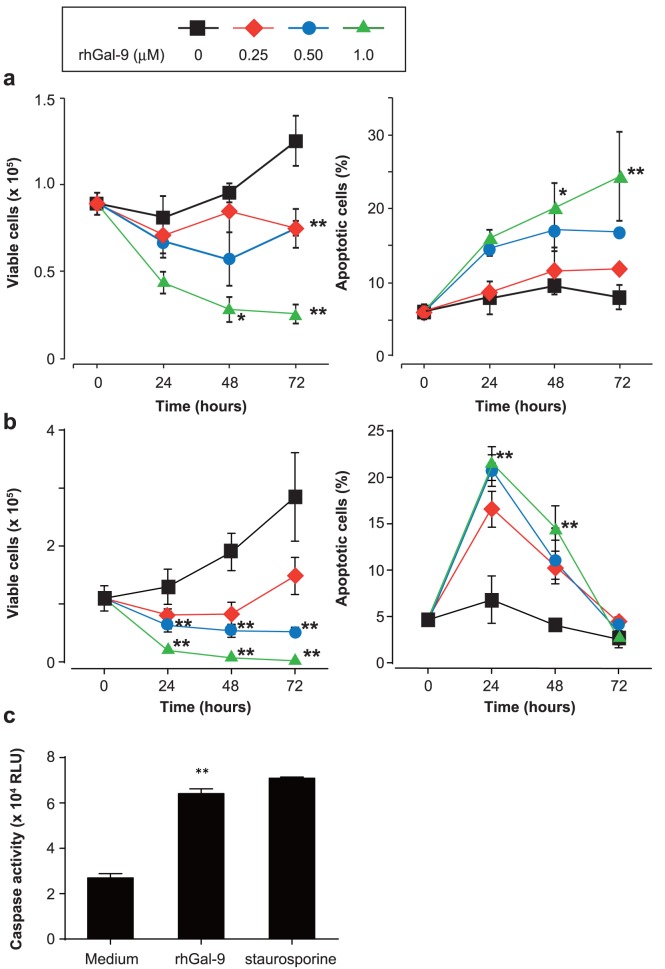
Galectin-9 induces apoptosis of HMC-1 cells. (a, b) HMC-1 cells pre-treated with (a) or without mitomycin C (b) were cultured in the presence of 0, 0.25, 0.5 or 1 µM recombinant human galectin-9 (rhGal-9) for the indicated time periods. The number of viable cells was determined by trypan blue staining. The proportion of propidium iodide-negative and annexin V-positive apoptotic cells was assessed by flow cytometry. (c) HMC-1 cells (no mitomycin C pre-treatment) were cultured in the presence and absence of 0.5 µM rhGal-9 or 0.1 µM staurosporine for 16 hours. Then the levels of caspase activity in the cells were determined. Data show the mean ± SD of triplicate samples and are a representative result of three independent experiments. *p<0.05 and **p<0.01 versus the corresponding values for the vehicle control.

We next examined the effects of Gal-9 on HMC-1 cell survival. After treatment with or without mitomycin C, HMC-1 cells were cultured in the presence and absence of 0.25, 0.5 and 1 µM rhGal-9 for 24, 48 and 72 hours. The number of trypan blue-negative viable cells was significantly decreased by 1 µM rhGal-9, while the percentage of propidium iodide-negative and annexin V-positive apoptotic cells was significantly increased irrespective of mitomycin C treatment (Figure 3a and 3b). In association with this, as in the case of staurosporine treatment, the levels of caspase-3/7 activity in HMC-1 cells were also significantly increased after rhGal-9 treatment (Figure 3c). These findings indicate that rhGal-9 induces apoptosis of HMC-1 cells.

### Gal-9 Inhibits Degranulation of HMC-1 Cells

**Figure 4:**
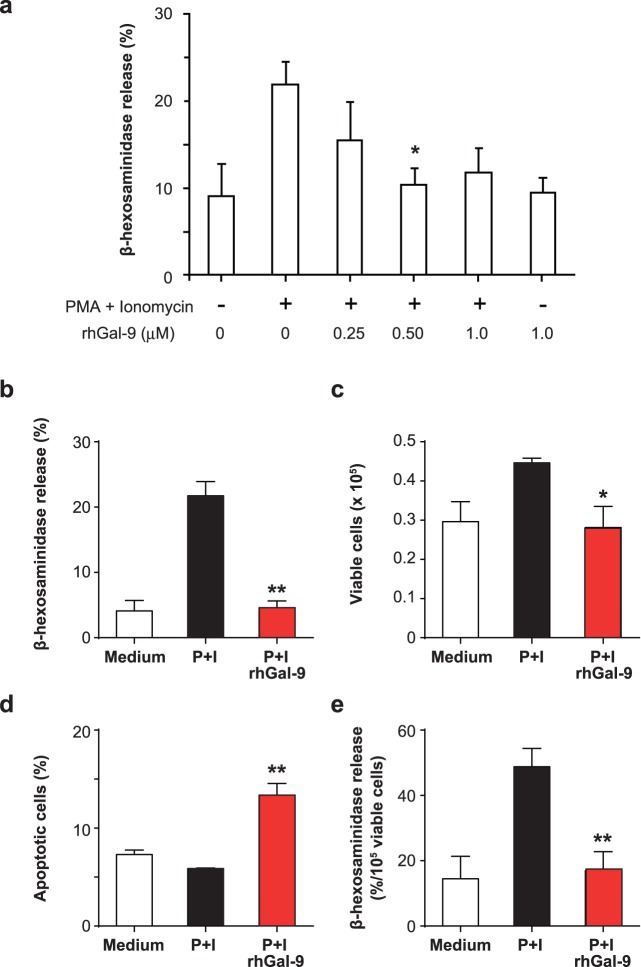
Galectin-9 inhibits PMA- and ionomycin-dependent degranulation of HMC-1 cells. (a, b) HMC-1 cells were treated with 0, 0.25, 0.5 or 1 µM (a) and 0 or 0.5 µM (b) recombinant human galectin-9 (rhGal-9) for 30 min. The cells were then stimulated with 0.1 µg/ml PMA +1 µg/ml ionomycin for 30 min. The level of degranulation was assessed from the activity of β-hexosaminidase in the culture supernatant and plotted as the percent release. (c) The number of viable cells in (b) was determined by trypan blue staining. (d) The proportion of propidium iodide-negative and annexin V-positive apoptotic cells in (b) was assessed by flow cytometry. (e) The relative level of degranulation per live HMC-1 cells was determined as (b)/(c). Data show the mean ± SD of triplicate samples and are a representative result of three (a) or two (b–e) independent experiments. *p<0.05, **p<0.01 versus PMA+ionomycin alone.

We next evaluated the effect of rhGal-9 on degranulation of HMC-1 cells. HMC-1 cells were treated with 0, 0.25, 0.5 and 1 µM of rhGal-9 for 30 min prior to stimulation with PMA+ionomycin. The level of degranulation, assessed by the release of β-hexosaminidase from the cells, was significantly suppressed by pretreatment with the optimal dose (0.5 µM) of rhGal-9 (Figure 4a). In the setting, pre-treatment with 0.5 µM rhGal-9 resulted in inhibition of cell survival (assessed as trypan blue-negative cells, and propidium iodide-negative and annexin V-positive apoptotic cells by flow cytometry) as well as degranulation after stimulation with PMA+ionomycin (Figure 4b–d), suggesting that the reduced degranulation of HMC-1 cells may be due to apoptosis of the cells after rhGal-9 treatment. On the other hand, the relative levels of degranulation per live HMC-1 cells were significantly reduced by pre-treatment with rhGal-9 (Figure 4e), suggesting that Gal-9 also inhibited PMA- and ionomycin-induced degranulation of HMC-1 cells independently of Gal-9-mediated apoptosis. Thus, these observations suggest that Gal-9 can inhibit PMA- and ionomycin-induced degranulation of HMC-1 cells, both directly and indirectly.

### Gal-9 Induces Cytokine Production by HMC-1 Cells

**Figure 5:**
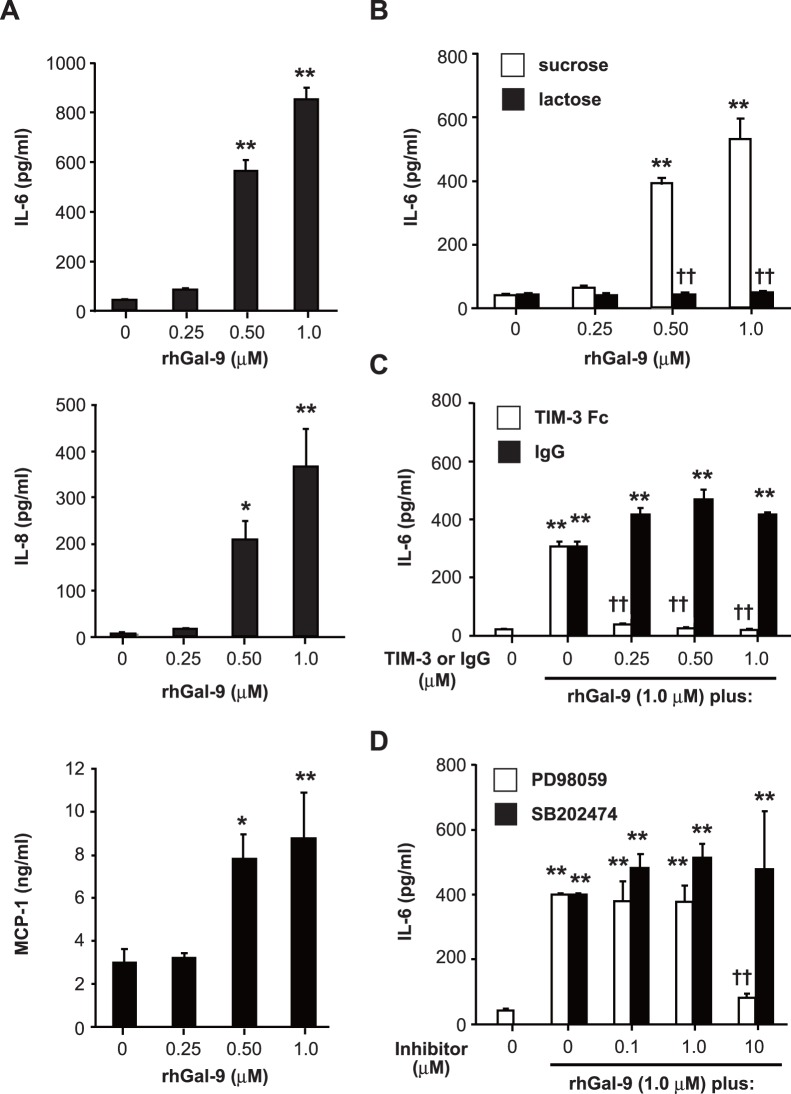
Gal-9 induces cytokine and chemokine production by HMC-1 cells. ELISA was performed to determine the levels of IL-6, IL-8 and MCP-1 in the culture supernatants of HMC-1 cells (**a**), HMC-1 cells pre-treated with 20 mM lactose or sucrose (**b**), HMC-1 cells pre-treated with recombinant human TIM-3/Fc (rhTIM-3/Fc) or control human IgG (human IgG) (**c**) and HMC-1 cells pre-treated with ERK inhibitor (PD98059) or its control (SB202474) (**d**) after 18 hours’ stimulation with 0, 0.25, 0.5 or 1 µM recombinant human Galectin-9 (rhGal-9). Data show the mean ± SD of triplicate samples and are a representative result of three independent experiments. *p<0.05 and/or **p<0.01 versus 0 µM rhGal-9 (**a–d**), and †p<0.05 and/or ††p<0.01 versus sucrose (**b**), control human IgG (**c**) or ERK inhibitor control (**d**).

In contrast to the inhibitory effect of rhGal-9 on degranulation, we found that IL-6, IL-8 and MCP-1 production by HMC-1 cells was dose-dependently induced by rhGal-9 (Figure 5a). It is known that most biological effects of galectins are mediated by their carbohydrate-binding activities. [5] In support of that, rhGal-9-mediated IL-6 production by HMC-1 cells was strongly suppressed by addition of an excessive amount of lactose but not sucrose (Figure 5b). Moreover, rhGal-9-mediated IL-6 production by HMC-1 cells was inhibited by addition of a soluble form of TIM-3 (rhTIM-3/Fc) but not control human IgG (Figure 5c), and by pre-treatment with PD98059 (an ERK1/2 inhibitor) but not SB202474 (control for the ERK1/2 inhibitor) (Figure 5d), suggesting that Gal-9-mediated ERK1/2 activation is required for cytokine production by HMC-1 cells.

## Discussion

In the present study, we demonstrated that Gal-9 has dual roles in the functions of a human mast cell line, HMC-1. That is, Gal-9 reduced survival by inducing apoptosis and suppressed degranulation in HMC-1 cells, while it induced cytokine and chemokine production by these cells by activating ERK1/2.

We show that Gal-9 induced phosphorylation of Erk1/2, but not p38 MAPK, in HMC-1 cells (Figure 2). On the other hand, however, Gal-9 induced maturation of human monocyte-derived DCs through activation of p38 MAPK, but not ERK1/2. [34] These observations suggest that the Gal-9-mediated signaling pathway may be different in distinct types of cells. Alternatively, the difference between DCs and HMC-1 cells may be mediated by distinct receptors such as TIM-3 and unknown molecules that interact with Gal-9. Indeed, the lectin property of Gal-9 was required for Gal-9-mediated cytokine production by HMC-1 cells (Figure 5b), but not by human DCs. [34] In addition, Gal-9 induced apoptosis of HMC-1 cells (Figure 3) as well as thymocytes, Th1 cells and Th17 cells in mice, and human melanoma cell lines. [2], [7], [21]–[23], [35] In contrast, we previously demonstrated that anti-TIM-3 antibody, which enhanced IgE/Ag-mediated cytokine production as an agonistic antibody, suppressed apoptosis of IL-3-induced mouse bone marrow cell-derived cultured mast cells. [16] These observations suggest that Gal-9-mediated responses may be dependent or independent of TIM-3, in different cells, since TIM-3 is also known to bind to phosphatidylserine. [36].

It was shown that Gal-9 bound IgE, resulting in inhibition of IgE/antigen-FcεRI-mediated degranulation in mouse mast cell lines by preventing IgE/antigen complex formation. [29] In the present study, because HMC-1 cells do not express FcεRI, [37] we assessed the effect of Gal-9 on PMA/ionomycin-mediated degranulation of HMC-1 cells. Figure 4 shows that Gal-9 suppressed that degranulation both directly and indirectly, suggesting that there might be distinct mechanisms underlying the inhibitory effects of Gal-9 on IgE/antigen-FcεRI-mediated and PMA/ionomycin-mediated mast cell degranulation.

Studies in rodents found that treatment with Gal-9 before antigen challenge resulted in attenuation of ovalbumin- and mite allergen-induced allergic airway inflammation as well as passive cutaneous anaphylaxis after antigen challenge. [27], [29] Gal-9′s attenuation of such disorders in mast cells [29] was due to suppression of degranulation, rather than induction of cytokines and chemokines, probably independent of TIM-3, since TIM-3-deficient mice normally developed allergic airway inflammation. [28] However, treatment with Gal-9 after antigen challenge may exacerbate inflammation in the late phase of allergic diseases by enhancing cytokine and chemokine production by mast cells and recruiting eosinophils to local inflammatory sites.

In conclusion, Gal-9 appears to play dual roles in the function of human mast cell line. Our results suggest that Gal-9 may modulate the induction and progression of allergic diseases by suppressing degranulation and enhancing cytokine and chemokine production of mast cells. In addition, Gal-9 may be a potential therapeutic target for immediate allergic reactions induced by mast cell degranulation.
